# Pharmacological Modulation of Injury-Induced Vascular Remodeling by Colchicine: An Integrated Experimental and Network-Based Analysis

**DOI:** 10.3390/biomedicines14051007

**Published:** 2026-04-28

**Authors:** Lutfi Cagatay Onar, Ersin Guner, Havva Nur Alparslan Yumun, Hasan Dindar, Ibrahim Yilmaz, Gunduz Yumun

**Affiliations:** 1 Department of Cardiovascular Surgery, Republic of Turkey, Ministry of Health, Dr. Ismail Fehmi Cumalioglu City Hospital, Tekirdag 59020, Turkey; 2 Department of Pharmacy, Republic of Turkey, Ministry of Health, Konya Numune Hospital, Konya 42060, Turkey; 3 Department of General Surgery, Republic of Turkey, Ministry of Health, Dr. Ismail Fehmi Cumalioglu City Hospital, Tekirdag 59020, Turkey; 4 Department of Pathology, Faculty of Medicine, Namık Kemal University, Tekirdag 59030, Turkey; 5 Unit of Pharmacovigilance, Republic of Turkey, Ministry of Health, Dr. Ismail Fehmi Cumalioglu City Hospital, Tekirdag 59020, Turkey

**Keywords:** colchicine, vascular remodeling, neointimal hyperplasia, vascular injury, histomorphometry, NF-κB signaling, molecular docking, protein–protein interaction network

## Abstract

**Background:** Colchicine is a microtubule-targeting anti-inflammatory agent with emerging relevance in cardiovascular disease; however, its effects on injury-induced vascular remodeling remain incompletely defined. **Methods:** In this study, a rat iliac artery clamp injury model was used to evaluate the effects of colchicine (0.5 mg/kg/day, oral gavage) over 28 days. Histomorphometric, histopathological, and immunohistochemical analyses were performed to assess vascular remodeling. In parallel, molecular docking and STRING/Cytoscape-based protein–protein interaction (PPI) network analyses were conducted to provide structural and systems-level context. **Results:** Colchicine significantly reduced intimal thickness, the intima-to-media (I/M) ratio, luminal stenosis, adventitial thickness, and collagen deposition, while preserving the lumen area and improving the remodeling index. Medial thickness was not significantly affected. Proliferative activity showed a decreasing trend without statistical significance. Circulating inflammatory cytokines, including TNF-α and IL-1β, did not differ significantly between groups. Docking analyses suggested potential interactions with β-tubulin, ADAM17, NLRP3, IKKβ, and RELA, while network analysis identified an interaction architecture centered on NF-κB-related regulatory components and inflammasome-associated signaling pathways. **Conclusions:** Colchicine attenuates injury-induced vascular remodeling in this experimental model. These findings, together with complementary in silico analyses, suggest a multi-target, inflammation-associated framework involving NF-κB-related and inflammasome-linked pathways. The in silico analyses provide supportive mechanistic context but do not establish causal relationships.

## 1. Introduction

Vascular remodeling following endothelial injury represents a major biological and clinical challenge, linking mechanical vascular trauma to luminal narrowing, restenosis, and ultimately long-term procedural failure. Rather than a purely proliferative process, injury-induced neointimal hyperplasia reflects a coordinated response involving endothelial disruption, inflammatory activation, vascular smooth muscle cell (VSMC) phenotypic switching, and extracellular matrix (ECM) remodeling, ultimately leading to structural reorganization of the vessel wall [1].

These processes are biologically interconnected: endothelial injury initiates inflammatory signaling, which promotes VSMC migration and dedifferentiation, leading to a synthetic phenotype characterized by increased extracellular matrix production and vessel wall thickening, ultimately compromising luminal patency [1,2]. Within this framework, neointimal growth should be considered part of a broader remodeling response involving medial and adventitial compartments, shaped by both mechanical stress and immune activation.

This concept is particularly relevant in vascular surgery and endovascular practice, where mechanical interventions such as clamping, anastomosis, and stenting can initiate vascular remodeling processes extending beyond the initial endothelial insult. The biological response to vascular injury is determined not only by the extent of endothelial denudation but also by the intensity and duration of subsequent inflammatory and reparative processes [1]. VSMC phenotypic switching from a contractile to a synthetic state is a key driver of neointimal expansion following vascular injury [2].

From a pharmacological perspective, these observations render inflammation-modulating agents particularly attractive in the context of vascular remodeling. Colchicine is a microtubule-disrupting alkaloid with a long-established role in the treatment of gout and autoinflammatory disorders, and its therapeutic relevance has increasingly expanded into cardiovascular medicine [3,4]. Its primary molecular action involves binding to tubulin, thereby interfering with microtubule polymerization and cytoskeletal dynamics [3,4]. Beyond cytoskeletal effects, colchicine modulates microtubule-dependent cellular processes, including leukocyte adhesion, chemotaxis, intracellular trafficking, and inflammatory signaling. Accordingly, it has been increasingly recognized as a pharmacological agent capable of attenuating inflammation-driven vascular risk [3,4,5]. This pharmacological profile supports its repositioning as a broader cardiovascular anti-inflammatory agent with potential translational relevance [3,4].

Despite this evolving role, an important pharmacological gap remains. Most clinical and translational data on colchicine in cardiovascular medicine focus on secondary prevention, plaque-associated inflammation, and a reduction in recurrent vascular events in atherosclerotic disease [3,4,5]. However, these contexts do not fully address whether colchicine can directly attenuate the structural vascular response to focal mechanical injury. This distinction is critical, as post-traumatic vascular remodeling represents a localized and temporally organized process involving endothelial loss, VSMC activation, cytokine signaling, and extracellular matrix remodeling within the injured vessel wall. Consequently, therapeutic effects observed in diffuse vascular inflammation may not necessarily translate into the suppression of intimal thickening, luminal stenosis, or structural remodeling following direct arterial injury. Evaluating colchicine in a controlled injury model is therefore essential to bridge the gap between systemic anti-inflammatory effects and structural vascular protection.

Several interconnected inflammatory pathways may contribute to injury-induced vascular remodeling, including NF-κB signaling, inflammasome activation, and cytokine-mediated vascular smooth muscle cell responses [5,6,7,8,9]. These mechanisms provide a biologically plausible framework linking inflammation to structural vascular changes and support evaluation of anti-inflammatory pharmacological interventions.

Within this framework, the pharmacological profile of colchicine becomes particularly relevant. Although its interaction with tubulin provides a primary molecular anchor, its biological effects extend to processes directly implicated in vascular injury, including leukocyte recruitment, inflammasome activity, and inflammatory signaling [3,4,5,6].

Integrative in silico approaches were used to provide supportive mechanistic context; however, these analyses do not establish causality and were interpreted conservatively to assess whether colchicine may plausibly interact with proteins implicated in vascular inflammation and remodeling.

Accordingly, the present study was designed to investigate whether colchicine mitigates injury-induced neointimal hyperplasia and vascular remodeling in a rat iliac artery clamp model. This model reproduces a clinically relevant form of focal vascular injury and enables quantitative assessment of structural outcomes related to vessel patency. By integrating histomorphometric and histopathological evaluation with molecular docking and STRING/Cytoscape-based PPI network analyses, the study provides a mechanistically informed preclinical assessment of colchicine in post-traumatic vascular remodeling. Within this framework, the experimental component addresses direct structural vascular effects, whereas the in silico component supports mechanistic plausibility within an inflammation-centered signaling context.

## 2. Materials and Methods

### 2.1. Animals and Experimental Design

All experimental procedures were approved by the Namik Kemal University Animal Experiments Local Ethics Committee (Approval No: T2021-609, Approval Date: 6 April 2021) and were conducted in accordance with institutional and national guidelines for the care and use of laboratory animals.

Adult male Wistar albino rats aged 4 months and weighing 250–300 g were included in the study. All animals were obtained from, housed in, and studied at the Namik Kemal University Experimental Animal Center. Male rats were selected to minimize hormonal variability and because their vascular dimensions are suitable for reproducible morphometric assessment in experimental vascular injury models.

Animals were maintained under standardized environmental conditions, including a 12 h light/dark cycle, ambient temperature of 23 ± 2 °C, and relative humidity of 50–60%. Standard laboratory chow and water were provided ad libitum throughout the study period.

To reduce biological variability, baseline characteristics such as body weight, snout-to-anus length, and proximal tail diameter were recorded prior to the experimental procedures. In addition, physiological parameters including heart rate and body temperature were monitored. Animals were housed under conditions designed to minimize stress-related variability, and care was taken to avoid overcrowding and potential dominance-related behavioral effects that could influence inflammatory responses.

Following an acclimatization period of 7–14 days, animals were randomly assigned to two experimental groups (*n* = 10 per group) using a computer-generated randomization sequence. Each animal was assigned a numerical identifier prior to allocation to ensure unbiased group assignment, and histological and morphometric assessments were performed by investigators blinded to group allocation.

Group 1 consisted of rats subjected to clamp-induced iliac arterial injury without pharmacological treatment. Group 2 consisted of rats subjected to the same clamp-induced arterial injury followed by colchicine treatment. In all animals, the contralateral iliac artery was preserved and used as an internal control to reduce inter-animal variability and allow for paired comparison within the same biological system. This approach has been used in vascular injury models to minimize inter-animal variability and reduce the number of animals required, while enabling paired morphometric comparison within the same physiological environment. Accordingly, the use of the contralateral artery as an internal paired control represents an established methodology allowing for a reduction in animal numbers while enabling direct within-animal comparison of remodeling responses.

The primary outcome measure was neointimal thickness, as assessed by histomorphometric analysis. No animals were excluded from the analysis. All animals completed the full 28-day experimental period without complications.

To ensure comparable handling conditions, rats in the untreated group received an equivalent volume of sterile saline administered using the same oral gavage technique as in the treatment group. All animals were monitored daily throughout the experimental period for general health status, behavioral changes, and potential adverse effects related to the intervention or experimental procedures.

**Figure 1:**
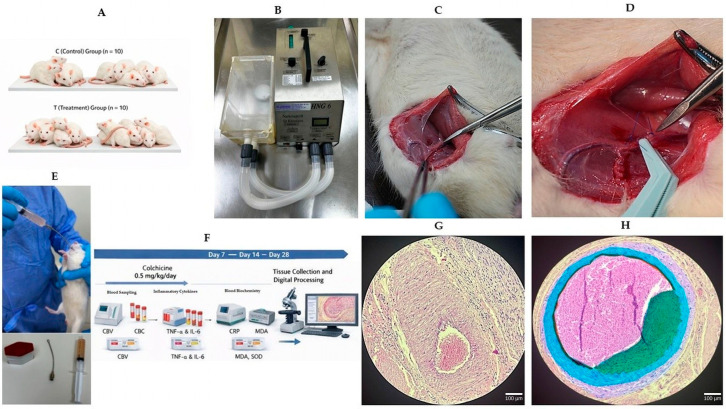
Experimental design of the rat iliac artery clamp injury model and colchicine treatment protocol. Schematic and representative images illustrating the experimental workflow. Adult male Wistar albino rats were subjected to clamp-induced iliac artery injury and then assigned to an untreated injury group receiving vehicle (saline) or a colchicine-treated group receiving colchicine (0.5 mg/kg/day, oral gavage) for 28 days. The study included a 28-day treatment period with serial blood sampling and final tissue collection. (**A**) Random allocation of rats into untreated injury (vehicle) and colchicine-treated groups (n = 10 per group). (**B**) The vascular clamp device used to induce iliac artery injury. (**C**) Surgical exposure of the iliac artery via a medial inguinal incision. (**D**) Bulldog clamp placement on the right iliac artery; Prolene sutures indicate the site of clamp-induced injury. (**E**) Oral gavage administration and blood sampling procedures. (**F**) The experimental timeline showing daily colchicine administration, blood sampling at days 7, 14, and 28, and tissue harvesting at day 28. (**G**) Representative histological sections of contralateral (internal control) and clamp-injured iliac arteries. (**H**) A representative processed histological image showing segmentation of vascular layers (lumen, intima, and media) used for morphometric analysis.

The overall experimental design and treatment timeline are summarized in Figure 1.

#### Colchicine Administration

Colchicine tablets (0.5 mg, Menarini, Florence, Italy) were finely crushed and freshly suspended in sterile saline immediately prior to administration to ensure homogeneity of the dosing solution. The drug was administered at a dose of 0.5 mg/kg/day.

Administration was performed once daily via oral gavage using an appropriate feeding needle, with the tip positioned in the distal third of the esophagus to ensure accurate delivery and consistent dosing.

Treatment was initiated immediately after the arterial clamping procedure and continued for 28 consecutive days throughout the remodeling period. The selected dose was based on commonly used experimental dosing ranges in cardiovascular research.

Animals in the untreated group received an equivalent volume of sterile saline administered using the same oral gavage technique to ensure comparable handling conditions between groups. All administrations were performed at the same time each day to reduce circadian variability.

Throughout the treatment period, animals were monitored daily for general health status, behavioral changes, and any signs of treatment-related adverse effects.

### 2.2. Iliac Artery Clamp Injury Model

A standardized iliac artery clamp injury model was used to induce controlled endothelial injury and initiate vascular remodeling. All surgical procedures were performed under inhalational anesthesia using sevoflurane delivered via a small-animal anesthesia system (HNG-6, GEWO Feinmechanik GmbH & Co. KG., Wörth, Germany). Anesthesia was induced with 4–5% sevoflurane and maintained at 2–3% in oxygen (flow rate: 1 L/min). Adequate anesthetic depth was confirmed by the absence of withdrawal reflexes prior to surgical manipulation. Body temperature was maintained using a thermostatically controlled heating pad, and animals were continuously monitored throughout the procedure.

Following sterile preparation of the surgical field, a 1 cm medial inguinal incision was performed to access the femoral vascular bundle. The superficial femoral artery was identified and carefully followed proximally to expose the iliac artery. The vessel was gently separated from surrounding connective tissue by blunt dissection, with care taken to preserve adjacent vascular and neural structures.

**Figure 2:**
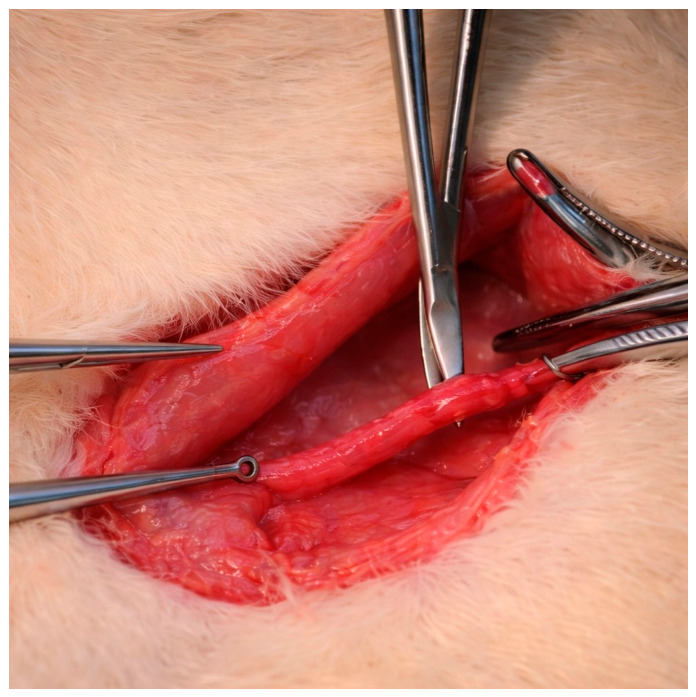
An intraoperative view of the iliac artery following blunt dissection and isolation prior to clamp application.

After adequate arterial mobilization, a microvascular plastic bulldog clamp (Vascu-Stat^®^, Scanlan International, Saint Paul, MN, USA) was applied to the iliac artery for 2 min to induce a standardized mechanical injury while preserving vascular continuity and avoiding full-thickness disruption. Following clamp removal, arterial patency was confirmed by direct visual inspection. The proximal and distal margins of the injured segment were marked with 7-0 polypropylene sutures (Prolene^®^, Ethicon, Somerville, NJ, USA) placed on the extra-adventitial surface to allow for precise identification of the injury site for subsequent histological and morphometric analyses (Figure 2).

The surgical incision was closed in anatomical layers using 4-0 polyglactin sutures (Pegleak^®^, Doğsan Medical, Istanbul, Turkey). Following surgery, animals were placed on a thermostatically controlled warming surface until full recovery from anesthesia and were subsequently returned to their cages under standard housing conditions. Postoperative monitoring included daily assessment of general behavior, mobility, wound condition, and any signs of procedure-related complications. The iliac artery was chosen because its caliber allows for reproducible surgical manipulation and reliable morphometric assessment in a medium-sized muscular artery [10,11].

### 2.3. Laboratory and Biochemical Analyses

Peripheral venous blood samples were collected on postoperative days 7, 14, and 28 to assess systemic inflammatory activity and oxidative stress during the vascular remodeling period. Blood samples were centrifuged immediately after collection, and serum aliquots were separated and stored at −80 °C until analysis to preserve biomarker stability.

Complete blood count parameters were measured using an automated hematology analyzer (VH5R Hemogram Analyzer, Hasvet Medikal A.S., Antalya, Turkey), which quantified standard hematological variables, including leukocyte subpopulations, erythrocyte indices, and platelet-related parameters.

Serum C-reactive protein (CRP) concentrations were determined using an immunoturbidimetric assay on the RX Monaco Clinical Chemistry Analyzer (Randox Laboratories Ltd., Crumlin, UK).

Circulating inflammatory cytokines were evaluated by measuring tumor necrosis factor-alpha (TNF-α) and interleukin-1 beta (IL-1β) levels using commercially available sandwich enzyme-linked immunosorbent assay (ELISA) kits (Elabscience, Biotechnology Inc., Houston, TX, USA; catalog no. E-EL-R2856 and E-EL-R0012), according to the manufacturers’ instructions.

Oxidative stress status was assessed by determining serum malondialdehyde (MDA) levels and superoxide dismutase (SOD) activity. MDA concentrations were quantified using a competitive ELISA kit (AssayGenie, Dublin Ireland; catalog no. RTEB1739), while SOD activity was measured using a colorimetric assay kit (Abbkine Scientific Co., Ltd., Wuhan, China; catalog no. KTE101023), following the manufacturers’ protocols.

All ELISA measurements were performed in duplicate to ensure analytical reliability. Optical density values were recorded at the recommended wavelengths using a microplate spectrophotometer (Epoch Microplate Spectrophotometer, BioTek Instruments, Winooski, VT, USA), and analyte concentrations were calculated from standard calibration curves generated for each assay.

### 2.4. Histopathological Examination

#### 2.4.1. Tissue Processing

Immediately after harvesting, arterial segments were immersed in 10% neutral buffered formalin and fixed for 24 h at room temperature to ensure optimal preservation of tissue architecture for subsequent histopathological, morphometric, and immunohistochemical analyses. Tissues were subsequently subjected to routine dehydration, paraffin embedding, and sectioning at 3 µm using standard histological procedures [12].

Sections were deparaffinized in xylene and rehydrated through graded ethanol solutions (100%, 80%, and 70%), followed by rinsing in distilled water.

For histological evaluation, sections were stained with hematoxylin–eosin (H&E) to assess general vascular morphology. Slides were stained with Harris hematoxylin for 8 min, rinsed under running water, differentiated in acid alcohol, washed again, and counterstained with eosin for 4 min. After dehydration through graded alcohols and clearing in xylene, sections were mounted with Entellan.

Additional sections were stained with Elastic Van Gieson (EVG) to visualize the elastic laminae and facilitate delineation of vascular compartments. Slides were incubated in alcoholic hematoxylin for 5 min, treated with 10% ferric chloride for 2 min, rinsed with distilled water, and exposed to Lugol’s iodine for 2 min before final washing, dehydration, clearing in xylene, and mounting.

Masson’s trichrome staining was performed to evaluate collagen deposition and ECM remodeling according to standard histological protocols.

All stained sections were examined using a Nikon Eclipse Ci light microscope (Nikon Corporation, Tokyo, Japan), and histological evaluation was performed in a blinded manner to minimize observer bias.

The intimal area was defined as the region between the luminal surface and the internal elastic lamina, which was more clearly delineated using EVG staining. Representative images from each specimen were obtained at 200× magnification.

#### 2.4.2. Morphometric Analysis

Digital histological images were acquired using a Nikon Eclipse Ci light microscope at a fixed magnification (×200) for all measurements.

For morphometric evaluation, vascular tissue layers, including the lumen, intima, and media, were manually delineated on digitized images using ImageJ software (version 1.54p, National Institutes of Health, Bethesda, MD, USA; https://imagej.org, accessed on 20 April 2026), including the Fiji distribution of ImageJ. All measurements were performed in a blinded manner by an independent investigator unaware of the experimental groups. Image calibration was performed using a stage micrometer prior to analysis.

For each specimen, five non-overlapping cross-sectional fields were analyzed, and mean values were used for statistical evaluation. Segmentation of vascular layers was performed based on morphological boundaries. Subsequently, pixel-based area quantification was conducted using MATLAB (version R2024b; MathWorks Inc., Natick, MA, USA) on calibrated images to ensure reproducible and standardized analysis, consistent with previously reported vascular morphometric analysis approaches [12].

Luminal stenosis was calculated as (1 − lumen area/IEL area) × 100 [12].

The remodeling index (RI) was calculated as the ratio of the external elastic lamina (EEL) area of the injured arterial segment to that of the contralateral non-clamped reference artery of the same animal. Values below 0.95 were interpreted as constrictive remodeling, whereas values approaching 1.0 indicated preservation of vessel geometry.

#### 2.4.3. Immunohistochemical Analysis

Immunohistochemical staining was performed to evaluate endothelial integrity, inflammatory cell infiltration, and cellular proliferation within the vascular wall. Sections were subjected to heat-induced antigen retrieval, followed by incubation with primary antibodies against CD68, CD3 and Ki-67 under conditions recommended by the manufacturers and optimized laboratory protocols. After washing, sections were incubated with appropriate secondary antibodies, and immunoreactivity was visualized using 3,3′-diaminobenzidine (DAB) as the chromogenic substrate. Sections were then counterstained with hematoxylin, dehydrated through graded alcohols, cleared in xylene, and mounted for microscopic evaluation.

Stained sections were examined under light microscopy. Proliferative activity was assessed by calculating the Ki-67 labeling index as the percentage of positively stained nuclei within the intimal region. CD68 and CD3 staining were used for qualitative assessment of inflammatory cell presence within the vascular wall and were not subjected to quantitative analysis; representative images are not presented as separate figure panels, as these markers served as descriptive rather than primary outcome measures.

### 2.5. In Silico Molecular Analysis Methods

Molecular docking analyses were performed to evaluate potential interactions of colchicine with protein targets relevant to vascular injury and inflammatory signaling. β-Tubulin was included as a reference target, while additional targets comprised ADAM17 (TACE), the NLRP3 inflammasome, an inhibitor of NF-κB kinase subunit beta (IKKβ), and RELA (NF-κB p65).

Molecular docking simulations were conducted using AutoDock 4.2.6, with protein and ligand preparation performed in AutoDockTools (MGLTools, version 1.5.6). Ligand preparation and file conversion steps were additionally performed using ChemDraw Ultra 12.0 and ChemBio3D Ultra 13.0 (PerkinElmer, Waltham, MA, USA), and Open Babel version 3.1.1 (Open Babel Project), as detailed in the Supplementary Materials. For each protein–ligand pair, at least 50 docking runs were performed using the Lamarckian Genetic Algorithm. The resulting conformations were evaluated based on predicted binding free energies (ΔG, kcal/mol), hydrogen bond interactions, and root mean square deviation (RMSD) values.

Binding poses with the lowest predicted binding free energies (ΔG, kcal/mol) and clustering RMSD values ≤ 2 Å were considered candidate docking conformations. Exploratory analyses of protein–ligand interactions, including hydrogen bonding and hydrophobic contacts, were performed within the MGLTools environment, and selected complexes were visualized in three-dimensional (3D) representations.

#### 2.5.1. Molecular Docking Analysis

Molecular docking analyses were performed to explore potential interactions of colchicine with proteins involved in inflammatory signaling and vascular remodeling processes associated with vascular injury. Target proteins representing key regulatory steps within the inflammatory cascade were selected. These targets were selected a priori to represent complementary levels of colchicine-related and injury-relevant biology, including the established tubulin-binding axis (β-tubulin), TNF-α processing (ADAM17), inflammasome activation (NLRP3), upstream NF-κB signaling (IKKβ), and downstream NF-κB transcriptional regulation (RELA). This biologically structured selection framework was intended to explore mechanistic plausibility within inflammatory pathways implicated in vascular remodeling.

β-Tubulin, the primary pharmacological target of colchicine, was included as a reference target. Additional targets comprised ADAM17 (TACE), a regulator of TNF-α shedding; the NLRP3 inflammasome, a mediator of inflammasome activation; and the inhibitor of NF-κB kinase subunit beta (IKKβ), a key kinase in the NF-κB signaling pathway. RELA (NF-κB p65), representing the transcriptionally active component of the NF-κB pathway, was also included to evaluate potential interactions at the level of downstream transcriptional regulation.

3D crystal structures of the target proteins were obtained from the Protein Data Bank (PDB; https://www.rcsb.org; accessed on 20 April 2026). Whenever available, structures containing co-crystallized ligands were preferentially selected. Docking protocol validation was performed by redocking co-crystallized ligands into their respective binding sites, yielding clustering RMSD values ≤ 2 Å and confirming accurate recovery of experimentally observed binding poses.

For each protein–ligand pair, at least 50 docking runs were performed using the Lamarckian Genetic Algorithm implemented in AutoDock, in accordance with the computational workflow described in the Supplementary Materials.

Docking analyses were conducted for the β-tubulin complex (PDB ID: 4O2B) [13], ADAM17 (PDB ID: 3E8R) [14], IKKβ (PDB ID: 4KIK) [15], the NLRP3 inflammasome (PDB ID: 7PZC) [16], and RELA (NF-κB p65) (PDB ID: 1LE9) [17]. 3D representations of the selected protein–ligand complexes, including hydrogen bonding and hydrophobic interactions, are provided in the Supplementary Materials.

#### 2.5.2. GO Enrichment and Network Topology Analyses

##### Protein–Protein Interaction (PPI) Network Construction and Functional Enrichment Analysis

An exploratory PPI network was constructed using the Search Tool for the Retrieval of Interacting Genes/Proteins (STRING) database (version 12.0; https://string-db.org; accessed on 20 April 2026) [18] for *Rattus norvegicus*. The primary seed set consisted of Tubb5, Adam17, Ikbkb, RELA, and Nlrp3, selected to explore cytoskeletal regulation together with TNF-related, NF-κB, and inflammasome signaling pathways potentially implicated in vascular remodeling.

Ikbkb and RELA were included as complementary components of the NF-κB signaling pathway, representing upstream kinase regulation and downstream transcriptional activity, respectively.

Network construction was performed using the STRING network with all available interaction evidence sources enabled, including text mining, experimental data, curated databases, co-expression, neighborhood, gene fusion, and co-occurrence. The minimum required interaction score was set to a high-confidence threshold (0.700) to increase confidence in the inferred interactions.

To expand the network while maintaining biological interpretability, the number of first-shell interactors was limited to 10, whereas second-shell interactors were excluded.

Functional enrichment analysis was performed using the integrated STRING enrichment tools. Enrichment categories included Gene Ontology Biological Process (GO-BP), GO Cellular Component (GO-CC), and Kyoto Encyclopedia of Genes and Genomes (KEGG) pathways. Statistical significance was assessed using false discovery rate (FDR) correction within the STRING platform.

The GO-BP enrichment profile was visualized using a bubble plot generated based on STRING enrichment output, in which bubble size reflects the number of genes associated with each biological process and color intensity represents statistical significance based on FDR values.

##### Network Visualization and Hub Gene Identification

The STRING-derived *Rattus norvegicus* PPI network was imported into Cytoscape software (version 3.10.4) for visualization and topological analysis [19]. Hub gene prioritization was performed using the cytoHubba plugin [20].

Nodes were ranked according to the Maximal Clique Centrality (MCC) algorithm, and the top 10 ranked nodes were identified as the candidate hub gene subset. For visualization, the network layout was arranged using a force-directed layout algorithm in Cytoscape.

To complement MCC-based prioritization, additional network topology parameters, including Degree, Betweenness Centrality, and Closeness Centrality, were evaluated to further characterize node connectivity, information flow, and network proximity [21]. These parameters were calculated within Cytoscape and used to assist hub gene identification.

### 2.6. Statistical Analysis

All statistical analyses were performed using IBM SPSS Statistics version 25.0 (IBM Corp., Armonk, NY, USA) and GraphPad Prism version 9.0 (GraphPad Software, San Diego, CA, USA). Continuous variables were assessed for normality using the Shapiro–Wilk test, supported by visual inspection of histograms and Q–Q plots. Normally distributed data were presented as the mean ± standard deviation (SD), whereas non-normally distributed data were reported as median with interquartile range (IQR). Categorical variables are expressed as counts and percentages.

Baseline biological and physiological parameters, including body weight, body length, tail diameter, body temperature, and heart rate, were compared between groups to assess baseline comparability prior to intervention using the independent samples t-test for normally distributed data or the Mann–Whitney U test for nonparametric data. Between-group comparisons for histomorphometric, histopathological, and biochemical variables were performed using the independent samples t-test for normally distributed variables and the Mann–Whitney U test for nonparametric variables. Ordinal histopathological variables were analyzed using the Mann–Whitney U test. Categorical variables were analyzed using Fisher’s exact test when appropriate.

Longitudinal biochemical measurements obtained on postoperative days 7, 14, and 28 were analyzed using a linear mixed-effects model with random intercepts. Time and treatment group were included as fixed effects, and individual animals were treated as random effects. Time-by-treatment interactions were evaluated to examine group differences in temporal changes.

Effect sizes were calculated to quantify the magnitude of between-group differences, using Cohen’s d for parametric data and rank-biserial correlation for nonparametric data. All statistical tests were two-tailed, and a *p*-value < 0.05 was considered statistically significant.

## 3. Results

### 3.1. Baseline Characteristics and Safety

Baseline biological and physiological characteristics were comparable between groups (Table 1).

Body weight, body length, tail diameter, body temperature, and heart rate did not differ significantly, suggesting adequate baseline homogeneity. No severe procedure-related complications were observed during the experimental period. A small number of animals in both groups exhibited mild weight loss during follow-up, with no apparent impact on general health status. All animals included in the analysis completed the study protocol.

Peripheral blood analyses performed on postoperative days 7, 14, and 28 did not show significant differences between groups in systemic inflammatory parameters. White blood cell counts and CRP levels showed a gradual decline over time, consistent with the resolution of the early postoperative inflammatory response.

No statistically significant differences were observed between groups for circulating inflammatory cytokines, including TNF-α and IL-1β (*p* > 0.05) at any time point.

In contrast, oxidative stress parameters showed time-dependent changes across both groups. MDA levels were highest during the early phase following vascular injury and decreased progressively over time, declining from 4.17 ± 0.56 nmol/mL on day 7 to 2.73 ± 0.41 nmol/mL by day 28. Conversely, SOD activity increased steadily during the remodeling phase, rising from 71.3 ± 9.4 U/mL on day 7 to 101.2 ± 11.3 U/mL at day 28.

Linear mixed-effects modeling suggested a significant time-dependent effect for both oxidative stress markers (*p* < 0.01). No significant group-by-time interaction was observed. No significant between-group differences were observed for oxidative stress parameters.

Detailed longitudinal peripheral blood data for inflammatory cytokines and oxidative stress parameters are provided in Supplementary Table S2.

### 3.2. Histomorphometric and Histopathological Outcomes

**Figure 3:**
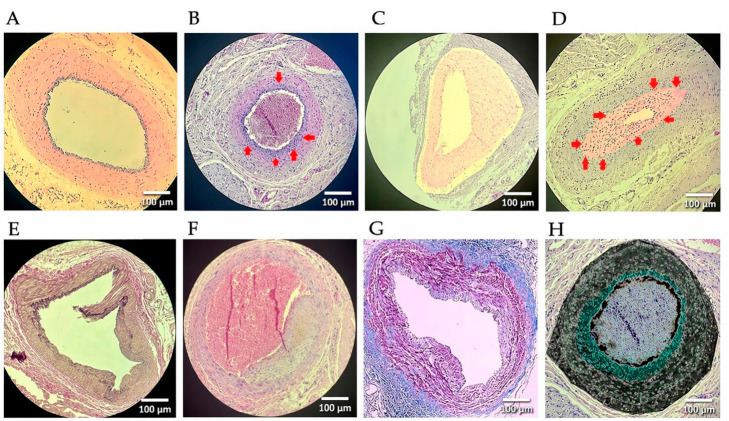
Representative histological and morphometric findings of iliac artery sections following clamp-induced injury and colchicine treatment. (**A**) A normal H&E-stained section of the contralateral, non-clamped iliac artery showing intact vascular architecture with clearly defined intima, media, and adventitia. (**B**) A H&E-stained section of a clamped artery demonstrating neointimal formation; arrows indicate the intimal layer. (**C**) A digitally processed H&E-stained image showing clear delineation of intimal and medial layers for morphometric assessment. (**D**) Pronounced neointimal hyperplasia with luminal narrowing and increased cellularity. Red arrows indicate the outer boundary of the intima and regions of concentrated proliferative cells. Ki-67 immunostaining showed a trend toward increased proliferative activity, without statistical significance. The image represents a digitally processed version generated using image processing software (ImageJ), in which intimal borders were enhanced to facilitate accurate thickness measurements and improved visualization of proliferative cell density. (**E**) A section from the colchicine-treated group showing focal intimal disruption with preserved endothelial lining (EVG). (**F**) Asymmetric neointimal thickening with luminal narrowing in a clamped artery. (**G**) A Masson’s trichrome-stained section showing collagen deposition within the vessel wall. (**H**) A representative processed image derived from panel (**B**) and used for pixel-based morphometric analysis, including quantification of tissue thickness, cellular density, and I/M ratio. Panels (**G**,**H**) are presented in a different format because they illustrate distinct methodological aspects of the analysis: panel (**G**) corresponds to Masson’s trichrome staining, whereas panel (**H**) represents a digitally processed image used for quantitative morphometric assessment. Quantitative morphometric analysis showed that intimal thickness was significantly lower in the colchicine-treated group (34 ± 10 µm vs. 60 ± 14 µm, *p* < 0.001), with a lower I/M ratio (0.32 ± 0.08 vs. 0.54 ± 0.12, *p* = 0.002). Medial thickness did not differ significantly between groups (*p* = 0.38).

Clamp-induced endothelial injury was associated with measurable vascular remodeling in both groups. Compared with the clamp-only group, the colchicine-treated group showed lower neointimal formation, larger lumen area, and reduced adventitial thickness (Table 2, Figure 3).

Lumen area was greater in the colchicine group (0.28 ± 0.06 mm^2^ vs. 0.18 ± 0.05 mm^2^, *p* = 0.003), with a reduction in luminal stenosis (18 ± 7% vs. 40 ± 9%, *p* < 0.001). Adventitial thickness was also lower in colchicine-treated animals (60 ± 13 µm vs. 82 ± 15 µm, *p* = 0.004). All image processing procedures were applied consistently across all groups.

The remodeling index was higher in the colchicine group (0.98 ± 0.05 vs. 0.89 ± 0.06, *p* = 0.006), suggesting a more preserved remodeling pattern. No statistically significant differences were observed between groups for medial area (*p* = 0.91), internal elastic lamina (IEL) area (*p* = 0.24), or external elastic lamina (EEL) area (*p* = 0.33).

Histological staining showed reduced ECM remodeling in the colchicine group, with lower collagen deposition and lower elastic lamina thickening.

Neointimal cellular proliferation was lower in the colchicine group based on Ki-67 values (2.2 ± 0.8% vs. 2.6 ± 0.9%), without statistical significance (*p* = 0.31).

Qualitative histological evaluation was consistent with these findings. Contralateral arteries exhibited preserved vascular architecture with clearly defined intimal, medial, and adventitial layers. In contrast, clamp-only arteries showed marked neointimal thickening, luminal narrowing, and increased cellularity.

**Figure 4:**
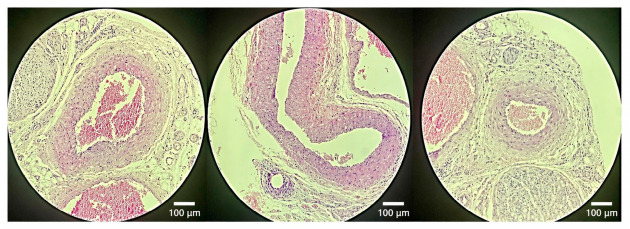
Representative histological sections from colchicine-treated animals (T2, T4, and T5). Sections show arterial wall morphology with limited neointimal formation and relatively preserved luminal architecture following clamp-induced injury. Original magnification: ×200.

In colchicine-treated animals, neointimal formation was lower and luminal architecture was relatively preserved. A more regular intimal and endothelial staining (H&E) pattern was consistent with better preservation of endothelial continuity in the treated specimens (Figure 3 and Figure 4).

### 3.3. Computerized Molecular Analysis

#### 3.3.1. Molecular Docking Findings

Molecular docking analyses were conducted to evaluate the potential binding interactions of colchicine with selected proteins implicated in inflammatory signaling pathways. The resulting conformations were clustered based on RMSD criteria, and representative docking poses from the dominant clusters were selected for further analysis. The conformation with the lowest predicted binding free energy (ΔG, kcal/mol) within the dominant cluster was used for further structural analysis. The results suggested that colchicine may interact with several proteins involved in inflammatory signaling pathways. Consistent with its established pharmacological target, colchicine exhibited predicted binding to β-tubulin (PDB ID: 4O2B), forming hydrogen bond interactions with Asn258 and Lys352 within the binding pocket.

For ADAM17 (PDB ID: 3E8R), colchicine formed two hydrogen bond interactions with Arg578 within a predicted binding region. Docking analysis of the NLRP3 structure (PDB ID: 7PZC) suggested a potential interaction involving Leu348 within the NACHT domain. Given the absence of a well-defined small-molecule binding pocket in the available NLRP3 structure, these findings should be interpreted as exploratory structural predictions.

Colchicine also showed a predicted interaction with IKKβ (PDB ID: 4KIK), involving a hydrogen bond interaction with Asp103.

Docking analysis of the RELA (NF-κB p65) structure (PDB ID: 1LE9) yielded a predicted binding free energy of −7.80 kcal/mol. Structural inspection of the docking pose suggested potential hydrogen-bond interactions involving Tyr36 and Lys123 residues. The predicted orientation positioned colchicine in proximity to the DNA-binding interface region of the RELA subunit.

**Figure 5:**
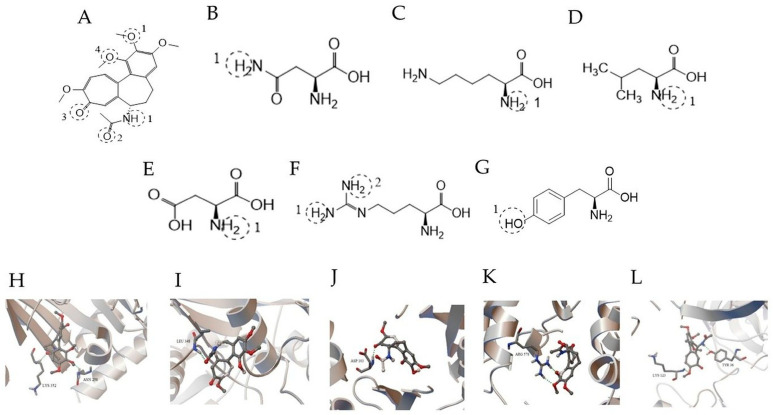
Predicted binding interactions of colchicine with selected inflammation-related target proteins. (**A**) Two-dimensional (2D) chemical structure of colchicine. (**B**–**F**) Two-dimensional (2D) structures of amino acid residues involved in hydrogen bonding interactions: (**B**) asparagine (ASN), (**C**) lysine (LYS), (**D**) leucine (LEU), (**E**) aspartic acid (ASP), and (**F**) arginine (ARG). Interaction sites are shown by dashed circles. (**G**) Representative two-dimensional (2D) structure of tyrosine (TYR) involved in RELA binding interactions. (**H**–**L**) Three-dimensional (3D) docking poses of colchicine with selected target proteins: (**H**) tubulin (PDB ID: 4O2B), (**I**) ADAM17 (PDB ID: 3E8R), (**J**) IKKβ (PDB ID: 4KIK), (**K**) NLRP3 (PDB ID: 7PZC), and (**L**) RELA (NF-κB p65) (PDB ID: 1LE9). Colchicine is depicted within the predicted binding region of each protein. Hydrogen bonds are represented by dashed lines, and interacting amino acid residues are labeled. Color coding represents different interaction types as defined by the visualization software.

It should be emphasized that docking results provide structural hypotheses rather than direct evidence of functional modulation. These findings are therefore interpreted as supportive in silico observations in the context of the experimental results (Table 3, Figure 5).

#### 3.3.2. STRING-Derived PPI Network and Functional Enrichment Analysis

**Figure 6:**
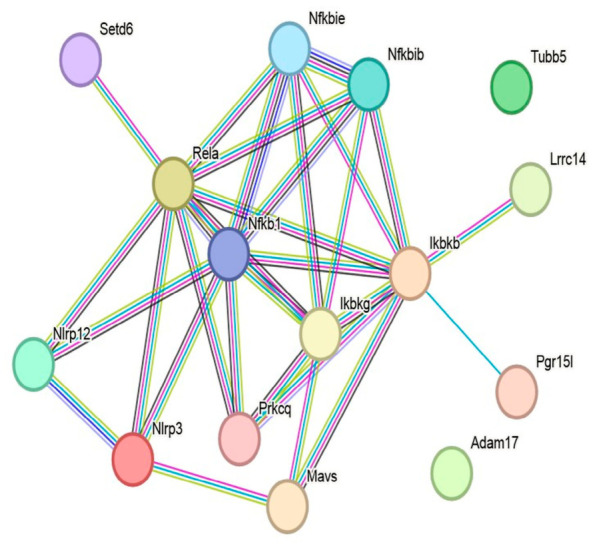
STRING-derived PPI network of colchicine-associated targets in *Rattus norvegicus*. The network was constructed using Tubb5, Adam17, Ikbkb, RELA, and Nlrp3 as seed proteins with a high-confidence interaction threshold (0.700) and a maximum of 10 first-shell interactors. The resulting network included an NF-κB-related regulatory module composed mainly of Ikbkb, RELA, Nfkb1, Ikbkg, Nfkbib, and Nfkbie, linked to an inflammasome-associated subnetwork centered on Nlrp3 and Nlrp12. In contrast, Adam17 and Tubb5 appeared as peripheral nodes under the selected confidence threshold and did not form direct edges in the final network layout. Edges represent protein–protein interactions. Line colors denote the type of interaction evidence, while edge thickness reflects the confidence level of each interaction.

STRING analysis yielded a PPI network consisting of 15 nodes and 30 edges (Figure 6). The network exhibited an average node degree of 4.0 and an average local clustering coefficient of 0.66. The expected number of edges for a random network of comparable size was 10, whereas the observed network contained significantly more interactions (PPI enrichment *p* = 5.39 × 10^−7^), suggesting that the selected seed proteins and their first-shell interactors form a non-random, biologically connected interaction network.

Topological inspection of the network suggested that the central interaction core was centered primarily on NF-κB-related regulatory components, including Ikbkb, RELA, Nfkb1, Ikbkg, Nfkbib, and Nfkbie, with additional connections to Prkcq and Mavs. An inflammasome-associated subnetwork centered on Nlrp3 and Nlrp12 was connected to this regulatory core, suggesting a structural linkage between NF-κB-related regulatory proteins and inflammasome-associated signaling components.

**Figure 7:**
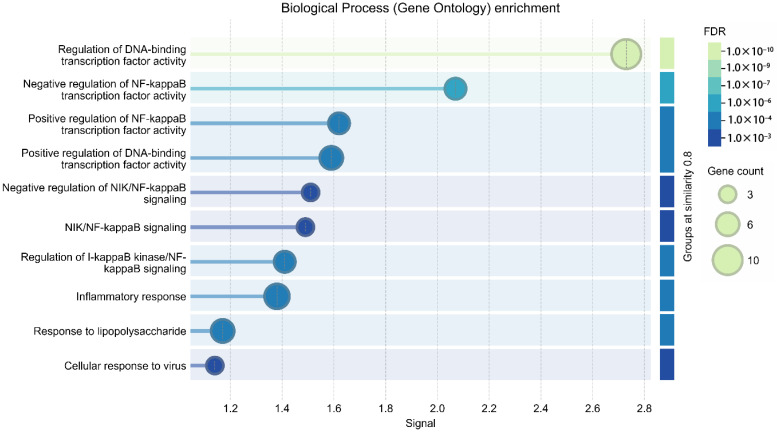
A GO-BP enrichment plot of the STRING-derived *Rattus norvegicus* network. The enriched biological processes were mainly related to NF-κB-related transcriptional regulation and inflammatory signaling, including regulation of DNA-binding transcription factor activity, regulation of I-κB kinase/NF-κB signaling, inflammatory response, and related immune regulatory processes. Bubble size represents gene count, whereas color intensity reflects statistical significance (FDR).

Functional enrichment analysis was consistent with an NF-κB-associated inflammatory signaling profile within the constructed network. Within the GO-BP category, the most significantly enriched terms included regulation of DNA-binding transcription factor activity (10 genes, FDR = 3.39 × 10^−10^), negative regulation of NF-κB transcription factor activity (5 genes, FDR = 1.98 × 10^−5^), inflammatory response (7 genes, FDR = 5.75 × 10^−5^), and positive regulation of NF-κB transcription factor activity (5 genes, FDR = 1.20 × 10^−4^). Additional enriched processes included regulation of IκB kinase (IKK)/NF-κB signaling, regulation of cytokine production, response to lipopolysaccharide, and negative regulation of NIK/NF-κB signaling (Figure 7, Table 4). Molecular function (MF) enrichment analysis is presented in Supplementary Figure S1.

Within the GO-CC category, the IκB kinase complex was significantly enriched (2 genes, FDR = 0.0363), in line with the central positioning of Ikbkb and Ikbkg within the interaction network.

KEGG pathway analysis showed enrichment of multiple inflammation-related pathways, including the NOD-like receptor signaling pathway (8 genes, FDR = 9.11 × 10^−12^), as well as NF-κB signaling, cytosolic DNA sensing, RIG-I-like receptor signaling, TNF signaling, Toll-like receptor signaling, and IL-17 signaling pathways.

Collectively, these findings suggest that the STRING-derived network is organized around NF-κB-associated regulatory components and is functionally linked to inflammasome-related signaling pathways.

#### 3.3.3. Cytoscape-Based Hub Gene Identification and Network Topology

To further characterize the topological organization of the interaction network beyond the initial STRING analysis, the rat PPI network was imported into Cytoscape (version 3.10.4) and analyzed using the cytoHubba plugin. Nodes were ranked according to the Maximal Clique Centrality (MCC) algorithm, and the ten highest scoring nodes were identified as hub genes.

The MCC-based ranking identified several components of the NF-κB signaling axis as the prominent nodes of the network. In particular, RELA, Nfkb1, Ikbkb, and Ikbkg exhibited the highest MCC scores, suggesting their central positions within the interaction network. Additional regulatory components of the NF-κB pathway, including Nfkbib and Nfkbie, were also among the top ranked nodes (Table 5).

To complement MCC-based hub prioritization, additional topological parameters were calculated using Cytoscape-based network analysis, including Degree, Betweenness Centrality, and Closeness Centrality. These metrics were used to provide complementary information regarding node connectivity, network communication flow, and relative proximity within the interaction structure.

Consistent with the MCC ranking, nodes within the NF-κB regulatory module exhibited higher connectivity and centrality values. RELA showed the highest betweenness centrality within the network, whereas proteins such as Adam17 and Tubb5 displayed low connectivity, in line with their peripheral positions in the interaction topology (Table 6).

**Figure 8:**
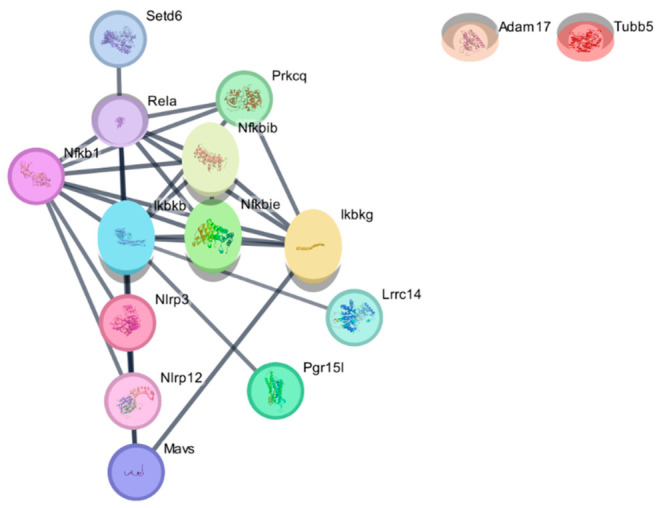
Cytoscape-based visualization of the STRING-derived PPI network in *Rattus norvegicus*. The STRING-derived interaction network was imported into Cytoscape (v3.10.4) for topological visualization and hub gene identification using the cytoHubba plugin (version 0.1). The network appeared to be organized around an NF-κB regulatory module composed of RELA, Nfkb1, Ikbkb, Ikbkg, Nfkbib, and Nfkbie. An inflammasome-associated subnetwork centered on Nlrp3 and Nlrp12 was connected to this regulatory module, with Mavs positioned as a downstream signaling component. Prkcq was located between NF-κB-related signaling elements. Adam17 and Tubb5 appeared as peripheral nodes under the selected interaction confidence threshold. Other first-shell interactors (Setd6, Lrrc14, and Pgr15l) were located at the network periphery and showed connections to the NF-κB-related module. Edges represent protein–protein interactions.

Visualization of the network topology suggested a modular organization composed of an NF-κB regulatory cluster connected to an inflammasome-associated subnetwork centered on Nlrp3 and Nlrp12 (Figure 8).

Additional proteins, including Prkcq and Mavs, were located between these modules, while other first-shell interactors (Setd6, Lrrc14, and Pgr15l) were positioned at the network periphery but showed connections with central signaling components.

Collectively, these findings suggest that the Cytoscape-derived interaction network is organized around NF-κB-associated regulatory components and is connected to inflammasome-related signaling elements.

## 4. Discussion

The present study suggests that colchicine may be associated with reduced injury-induced vascular remodeling in a rat iliac artery clamp model. This pattern was characterized by lower neointimal formation, reduced luminal stenosis, decreased adventitial thickness, and lower cellular proliferation, together with relatively preserved lumen area. These findings are consistent with a potential influence of colchicine on vascular structural changes following mechanical endothelial injury. Collectively, the observations support the hypothesis that colchicine may contribute to modulation of injury-driven vascular remodeling processes, although these results should be interpreted as exploratory and hypothesis-generating.

The clamp-induced iliac artery injury model produces controlled endothelial disruption and vascular wall injury, followed by inflammatory activation and reparative remodeling responses. Vascular remodeling after endothelial damage is a complex and dynamic process involving inflammatory activation, VSMC migration and proliferation, and ECM reorganization [1,5,6,9]. Previous studies have shown that endothelial denudation triggers a cascade of events involving cytokine release, NF-κB activation, and phenotypic switching of VSMCs, ultimately leading to neointimal hyperplasia and vessel wall thickening [2,7,9,11]. In this context, the observed reduction in intimal thickness, the I/M ratio, and proliferative indices in the present study may be consistent with a potential influence of colchicine on processes involved in pathological remodeling, rather than nonspecific effects on vessel structure.

Colchicine is a well-established microtubule-targeting agent with anti-inflammatory properties, and its cardiovascular effects have been increasingly recognized in recent years [3,4,13]. Mechanistically, colchicine disrupts microtubule polymerization, which may influence intracellular trafficking, inflammasome assembly, and leukocyte activation. Consistent with these known effects, the present study showed lower neointimal cellular proliferation, as reflected by mildly decreased Ki-67 expression, together with reduced ECM remodeling. These findings are in line with prior reports suggesting that colchicine may modulate vascular inflammation and cellular turnover in injury-related settings.

Interestingly, systemic inflammatory markers, including TNF-α and IL-1β, did not differ significantly between groups, whereas histological and morphometric changes within the vascular wall were pronounced. This apparent discrepancy may be consistent with the possibility that the observed effects of colchicine reflect local vascular inflammatory responses rather than systemic cytokine suppression, although this interpretation remains inferential in the absence of direct tissue-level molecular quantification. A similar dissociation between systemic and local inflammatory responses has been described in experimental vascular injury models, where localized signaling within the vessel wall may contribute to remodeling outcomes [5,6]. This pattern may indicate that colchicine-related effects occur predominantly at the vascular tissue level, which could explain the absence of detectable changes in circulating inflammatory markers.

To provide exploratory structural and systems-level context for these findings, in silico analyses were performed. Molecular docking suggested potential interactions of colchicine with multiple proteins involved in inflammatory signaling, including β-tubulin, ADAM17, NLRP3, IKKβ, and RELA. While these interactions cannot be interpreted as direct evidence of functional inhibition, they are consistent with the established role of colchicine in modulating microtubule dynamics and inflammation-related pathways. In particular, the predicted interaction with β-tubulin is compatible with the known pharmacological target of colchicine, whereas interactions with NLRP3 and NF-κB-related components are in line with pathways previously implicated in vascular inflammation and remodeling [6,7,8].

Complementary network analysis using STRING and Cytoscape further suggested that the selected targets are embedded within an interaction architecture related to NF-κB-associated regulatory components and connected to inflammasome-associated signaling pathways. Enrichment of pathways such as NF-κB signaling, NOD-like receptor signaling, and TNF signaling is consistent with the possibility that the observed in vivo effects occur within a broader inflammation-associated signaling framework. Importantly, these analyses are not intended to define a single mechanistic pathway but rather to provide a systems-level context that is consistent with the experimental observations. These complementary in silico analyses were included to provide mechanistic plausibility and systems-level context for the experimental observations rather than to establish causal molecular mechanisms. Future studies may consider an alternative strategy based on overlapping gene sets between colchicine-associated targets and injury-induced vascular remodeling-related genes; the present exploratory framework intentionally prioritized hypothesis-driven target selection followed by network-based contextualization.

The enrichment of both positive and negative regulation of NF-κB transcription factor activity reflects the regulatory complexity of this pathway, indicating modulation rather than a strictly directional activation or inhibition.

The absence of Tubb5 or ADAM17 among the top ranked hub nodes does not preclude their biological relevance, as the network topology analysis prioritizes connectivity rather than pharmacological targeting. These proteins were included as seed nodes to provide biological context linking colchicine-related pathways to inflammatory signaling within vascular remodeling.

Taken together, the findings of the present study are consistent with the possibility that colchicine may be associated with reduced vascular remodeling through modulation of inflammation-associated processes related to proliferative, structural, and ECM-related pathways. Rather than acting through a single linear mechanism, the combined in vivo and in silico results may be compatible with a multi-target framework including NF-κB-related regulatory components and inflammasome-linked signaling.

In the context of the existing literature, the present study provides experimental observations suggesting that colchicine may be associated with reduced injury-induced vascular remodeling in a controlled clamp-induced arterial injury model, extending its potential pharmacological relevance beyond atherosclerosis-related contexts to mechanically driven vascular injury. While previous work has primarily examined colchicine in atherosclerosis or stent-related models, the current study evaluates its effects in a controlled clamp-induced arterial injury model, enabling assessment of injury-driven remodeling processes. In addition, the integration of quantitative morphometric data with histopathological and immunohistochemical analyses offers a multi-level evaluation of vascular structure. The incorporation of molecular docking and network-based approaches situates these observations within a broader structural and systems-level context.

Several limitations should be acknowledged. First, although the in vivo findings are consistent with the histological and morphometric level, direct molecular validation of signaling pathways within vascular tissue was not performed. Second, the in silico analyses involve a degree of abstraction. Molecular docking was conducted using structurally available protein models, which predominantly consist of human-derived crystal structures due to the limited availability of high-resolution rat protein structures suitable for ligand-binding analysis, whereas network and enrichment analyses were performed in *Rattus norvegicus* to match the experimental model. Although this cross-species approach is commonly used to balance structural accuracy and biological relevance, it does not establish species-identical molecular interactions and should therefore be interpreted with caution.

Third, the network-based analyses were designed as exploratory contextual tools and were not intended to generate model-specific mechanistic targets from the current experimental system. Accordingly, these analyses provide supportive systems-level context but do not substitute for direct transcriptomic approaches such as RNA sequencing (RNA-seq) or single-cell transcriptomic profiling. In addition, the immunohistochemical panel was limited to markers of inflammatory cell presence and cellular proliferation, and pathway-specific markers such as VCAM-1 (an NF-κB target), cleaved caspase-1 (p20), and gasdermin D (GSDMD) were not assessed. Finally, the molecular docking findings represent structural predictions and were not supported by biophysical validation methods such as surface plasmon resonance (SPR), isothermal titration calorimetry (ITC), or thermal shift assays, as well as functional activity assays, which would be required to confirm direct target engagement.

Furthermore, the experimental findings were obtained in a rat model, and caution is required when extrapolating these results to human vascular biology. While preclinical models provide controlled conditions for mechanistic investigation, species-specific differences in inflammatory responses, tissue repair dynamics, and pharmacological sensitivity may influence translational applicability. Previous experimental studies in different tissue contexts have also highlighted that biological responses observed in animal models may not fully reflect those in human systems [22,23]. Furthermore, histological assessments, although performed in a blinded manner, inherently involve a degree of observer-dependent interpretation, which may introduce subtle variability.

Finally, although the sample size allowed for the detection of morphometric differences, it may limit the identification of more subtle systemic effects.

## 5. Conclusions

The present study demonstrates that colchicine attenuates injury-induced vascular remodeling in a rat arterial clamp model, with consistent effects on neointimal formation, cellular proliferation, and ECM remodeling. These findings, supported by complementary in silico analyses, suggest a multi-target, inflammation-associated framework that involves NF-κB-related and inflammasome-linked signaling pathways. Further studies integrating molecular validation and translational models are warranted to better define the mechanistic basis of these observations.

## Figures and Tables

**Table 1:** Baseline physiological characteristics of control and colchicine-treated rats (*n* = 10 per group).

Rat ID	Weight (g)	Length (cm)	Tail Diameter (mm)	Body Temp (°C)	Heart Rate (bpm)	Hierarchy
Control group						
C1	268	21.4	4.8	37.3	356	So
C2	275	22.1	5.0	37.5	362	So
C3	282	22.5	5.1	37.4	348	Do
C4	260	21.0	4.7	37.2	360	So
C5	271	21.8	4.9	37.6	352	So
C6	289	22.7	5.2	37.5	358	So
C7	254	20.9	4.6	37.3	366	So
C8	276	22.0	5.0	37.4	355	So
C9	267	21.6	4.8	37.2	349	So
C10	284	22.3	5.1	37.5	361	So
Mean ± SD	272.6 ± 10.9	21.83 ± 0.61	4.92 ± 0.19	37.39 ± 0.14	356.7 ± 5.8	1 dominant
Colchicine group						
T1	272	21.9	4.9	37.4	354	So
T2	286	22.6	5.2	37.6	360	So
T3	263	21.3	4.7	37.3	357	So
T4	277	22.0	5.0	37.5	351	So
T5	291	22.8	5.3	37.4	347	Do
T6	269	21.7	4.8	37.2	359	So
T7	258	21.1	4.7	37.3	364	So
T8	283	22.4	5.1	37.5	353	So
T9	274	21.9	4.9	37.4	358	So
T10	266	21.5	4.8	37.2	356	So
Mean ± SD	273.9 ± 10.5	21.92 ± 0.55	4.94 ± 0.21	37.38 ± 0.13	355.9 ± 4.9	-

So: subordinate; Do: dominant.

**Table 2:** Morphometric and histological parameters in the clamp-only and clamp + colchicine groups (*n* = 10 per group).

Parameter	Clamp Only (*n* = 10)	Clamp + Colchicine (*n* = 10)	*p* Value
Intimal thickness (µm)	60 ± 14	34 ± 10	<0.001
Medial thickness (µm)	112 ± 16	106 ± 14	0.38
Intima/media ratio	0.54 ± 0.12	0.32 ± 0.08	0.002
Lumen area (mm^2^)	0.18 ± 0.05	0.28 ± 0.06	0.003
Intimal area (mm^2^)	0.12 ± 0.03	0.06 ± 0.02	0.001
IEL area (mm^2^)	0.30 ± 0.07	0.34 ± 0.08	0.24
Medial area (mm^2^)	0.20 ± 0.04	0.20 ± 0.04	0.91
EEL area—injured (mm^2^)	0.50 ± 0.09	0.54 ± 0.10	0.33
EEL area—contralateral reference (mm^2^)	0.56 ± 0.07	0.55 ± 0.06	0.74
Luminal stenosis (%)	40 ± 9	18 ± 7	<0.001
Adventitial thickness (µm)	82 ± 15	60 ± 13	0.004
Remodeling index	0.89 ± 0.06	0.98 ± 0.05	0.006
Collagen deposition (µm)	60 ± 15	35 ± 12	0.002
Elastic lamina thickening (µm)	6.0 ± 1.5	4.0 ± 1.2	0.005
Ki-67 proliferation index (%)	2.6 ± 0.9	2.2 ± 0.8	0.31

Values are expressed as the mean ± SD. Data distribution was assessed prior to analysis; parametric or nonparametric tests were applied accordingly. Between-group comparisons were performed using the independent samples *t*-test for normally distributed variables or the Mann–Whitney U test for non-normally distributed variables, as determined by the Shapiro–Wilk test. *p* < 0.05 was considered statistically significant. The remodeling index was calculated as injured EEL area divided by contralateral non-clamped reference EEL area obtained from the same animal. Luminal stenosis (%) was calculated as (1 − lumen area/IEL area) × 100.

**Table 3:** Predicted binding affinities and hydrogen bonds between colchicine and selected target proteins identified by molecular docking.

Compound	Target Protein	PDB ID	Binding Energy (kcal/mol)	Clustering RMSD (Å)	Hydrogen Bonds (Distance Å)
Colchicine	Tubulin	4O2B	−10.90	0.23	O-1 (Colchicine)—H-1 (Asn258) (1.99 Å); O-2 (Colchicine)—H-1 (Lys352) (2.23 Å)
Colchicine	NLRP3	7PZC	−8.97	0.11	O-2 (Colchicine)—H-1 (Leu348) (2.15 Å)
Colchicine	IKKβ	4KIK	−8.30	0.47	O-3 (Colchicine)—H-1 (Asp103) (1.89 Å)
Colchicine	ADAM17	3E8R	−10.97	0.05	O-1 (Colchicine)—H-1 (Arg578) (1.78 Å); O-4 (Colchicine)—H-2 (Arg578) (2.21 Å)
Colchicine	RELA	1LE9	−7.80	0.37	Colchicine H-1—O-1 (Tyr36) (2.07 Å); Colchicine O-1—H-1 (Lys123) (2.13 Å)

**Table 4:** Functional enrichment analysis of the STRING-derived *Rattus norvegicus* PPI.

Category	Term/Pathway	Gene Count	FDR
GO Biological Process	Regulation of DNA-binding transcription factor activity	10	3.39 × 10^−10^
GO Biological Process	Negative regulation of NF-κB transcription factor activity	5	1.98 × 10^−5^
GO Biological Process	Inflammatory response	7	5.75 × 10^−5^
GO Biological Process	Positive regulation of DNA-binding transcription factor activity	6	5.75 × 10^−5^
GO Biological Process	Positive regulation of NF-κB transcription factor activity	5	1.20 × 10^−4^
GO Biological Process	Regulation of I-κB kinase/NF-κB signaling	5	3.00 × 10^−4^
GO Biological Process	Regulation of cytokine production	7	3.60 × 10^−4^
GO Biological Process	Response to lipopolysaccharide	6	3.80 × 10^−4^
GO Biological Process	Negative regulation of NIK/NF-κB signaling	3	7.10 × 10^−4^
GO Cellular Component	IκB kinase complex	2	0.0363
KEGG Pathway	NOD-like receptor signaling pathway	8	9.11 × 10^−12^
KEGG Pathway	Cytosolic DNA-sensing pathway	6	6.92 × 10^−11^
KEGG Pathway	RIG-I-like receptor signaling pathway	6	1.20 × 10^−10^
KEGG Pathway	NF-κB signaling pathway	5	1.17 × 10^−7^
KEGG Pathway	TNF signaling pathway	4	7.72 × 10^−6^
KEGG Pathway	Toll-like receptor signaling pathway	4	4.50 × 10^−6^
KEGG Pathway	IL-17 signaling pathway	4	4.06 × 10^−6^

**Table 5:** Top hub genes identified by cytoHubba MCC analysis in Cytoscape network.

Rank	Gene Symbol	MCC Score
1	RELA	151
2	Nfkb1	150
3	Ikbkb	148
4	Ikbkg	146
5	Nfkbib	120
6	Nfkbie	120
7	Prkcq	24
8	Nlrp3	7
9	Nlrp12	6
10	Mavs	3

**Table 6:** Topological properties of the Cytoscape-derived *Rattus norvegicus* PPI network.

Gene Symbol	Degree	Betweenness Centrality	Closeness Centrality
RELA	9	70.0	0.56839
Nfkb1	8	32.0	0.46652
Ikbkb	7	48.0	0.51223
Ikbkg	7	16.0	0.51223
Nfkbib	5	0.0	0.64826
Nfkbie	5	0.0	0.64826
Prkcq	4	0.0	0.56839
Nlrp3	4	8.0	0.46346
Nlrp12	3	0.0	0.46346
Mavs	2	8.0	0.30779

## Data Availability

The data presented in this study are available in the article and Supplementary Materials. The in vivo experimental data are included within the manuscript, while the In Silico analyses were performed using publicly available datasets. Processed data supporting the findings of this study are available from the corresponding author on request.

## Supplementary Materials

The following supporting information can be downloaded at: https://www.mdpi.com/article/10.3390/biomedicines14051007/s1. Supplementary Methods: Detailed Molecular Docking Workflow; Figure S1: Molecular Function (MF) Gene Ontology enrichment analysis of the selected target genes. Terms are ranked based on adjusted p-values, and the network visualization reflects functional similarity clustering; Table S1: Grid parameters used for molecular docking simulations. For each target protein, the docking grid box was defined to encompass the functional binding region or active site of the respective structure. Grid size and spacing parameters were kept constant across all docking simulations; Table S2: Longitudinal peripheral blood parameters in control and colchicine-treated groups.

## Abbreviations

The following abbreviations are used in this manuscript:

ADAM17	A disintegrin and metalloproteinase 17
BP	Biological process
CRP	C-reactive protein
ECM	Extracellular matrix
ELISA	Enzyme-linked immunosorbent assay
GO	Gene Ontology
GO-BP	Gene Ontology Biological Process
I/M	Intima-to-media ratio
IKKβ	Inhibitor of nuclear factor kappa-B kinase subunit beta
IL-1β	Interleukin-1 beta
KEGG	Kyoto Encyclopedia of Genes and Genomes
MDA	Malondialdehyde
MCC	Maximal Clique Centrality
NF-κB	Nuclear factor kappa-B
NLRP3	NLR family pyrin domain containing 3
PPI	Protein–protein interaction
RELA	v-Rel avian reticuloendotheliosis viral oncogene homolog A
RMSD	Root mean square deviation
SOD	Superoxide dismutase
TNF-α	Tumor necrosis factor alpha
VSMC	Vascular smooth muscle cell
