# Supplementary material for: Pharmacological Modulation of Injury-Induced Vascular Remodeling by Colchicine: An Integrated Experimental and Network-Based Analysis
Source: Biomedicines. 2026 Apr 28;14(5):1007. doi: 10.3390/biomedicines14051007 (PMC13203723; doi:10.3390/biomedicines14051007)
Supplement: Supplementary file 1 [file biomedicines-14-01007-s001.zip › biomedicines-4241728-supplementary.pdf]

Supplementary Materials

Title Pharmacological Modulation of Injury-Induced Vascular Remodeling by Colchicine: An Integrated Experimental and Network-Based Analysis

Supplementary Materials Overview

This Supplementary Materials file provides additional methodological details and supporting data relevant to the analyses presented in the main manuscript. It includes the detailed molecular docking workflow, supplementary Gene Ontology molecular function enrichment results, and longitudinal peripheral blood parameters added to improve transparency, reproducibility, and completeness of reporting. These supplementary materials are intended to support the interpretation of the main findings and should not be considered independent mechanistic validation.

Supplementary Contents

- Figure S1. Molecular Function (MF) Gene Ontology enrichment analysis

- Table S2. Longitudinal Peripheral Blood Parameters in Control and Colchicine-Treated Groups

- Supplementary Methods. Detailed Molecular Docking Workflow

Supplementary Methods and Additional Data

Detailed Molecular Docking Workflow

This supplementary document provides a complete and reproducible description of the molecular docking workflow referenced in the main manuscript. The objective of these analyses was exploratory—to evaluate mechanistic plausibility rather than establish causality—by assessing potential interactions of colchicine with selected vascular remodeling and inflammatory targets.

1. Software Environment

All molecular docking analyses were performed using:

- AutoDock 4.2.6 for docking simulations
- AutoDockTools (MGLTools) version 1.5.6 for receptor–ligand preparation and grid definition

- Open Babel version 3.1.1 for file conversion
- ChemDraw Ultra 12.0 and ChemBio3D Ultra 13.0 for ligand construction and energy minimization
- Python Molecular Viewer (PMV) 1.5.6 for visualization and interaction inspection

Docking simulations were executed using the Lamarckian Genetic Algorithm implemented in AutoDock.

2. Ligand Preparation

Colchicine chemical structures were generated using ChemDraw Ultra 12.0. Three-dimensional conformations were produced and subjected to energy minimization using the Merck Molecular Force Field (MMFF94) in ChemBio3D Ultra 13.0.

- The lowest energy conformers were exported in PDB format and converted to PDBQT format using Open Babel (v3.1.1).
- During ligand preparation:
    - Gasteiger charges were assigned
    - Rotatable bonds were defined automatically
    - Polar hydrogens were added

These prepared ligand files were used for all subsequent docking analyses.

3. Receptor Preparation (General Procedure)

Protein structures were obtained from the Protein Data Bank (https://www.rcsb.org). For all receptors, identical preprocessing steps were applied to ensure methodological consistency:

1. Removal of crystallographic water molecules
2. Removal of co-crystallized ligands or inhibitors
3. Addition of polar hydrogen atoms
4. Assignment of Kollman charges
5. Conversion to PDBQT format using AutoDockTools

Docking grid boxes were centered on experimentally validated ligand binding regions whenever available.

For each receptor–ligand pair:

- At least 50 independent docking runs were performed per receptor–ligand pair, from which the top-ranked poses (≥10 conformations) were selected based on binding energy and RMSD clustering criteria.
- Lamarckian Genetic Algorithm parameters were kept consistent across targets
- Conformations with the lowest binding energy and RMSD ≤ 2 Å were considered stable docking solutions

4. Table S.1. Grid parameters used for molecular docking simulations. For each target protein, the docking grid box was defined to encompass the functional binding region or active site of the respective structure. Grid size and spacing parameters were kept constant across all docking simulations.

Target Protein	PDB ID	Functional Domain / Binding Region	Grid Center (Å)	Grid Size (Å)	Grid Spacing (Å)
β-Tubulin	4O2B	α/β tubulin heterodimer; colchicine-binding site	x=16.056, y=64.861, z=46.056	40×40×40	0.375
ADAM17	3E8R	Catalytic metalloprotease domain (Zn^{2+} present)	x=7.917, y=6.972, z=28.361	40×40×40	0.375
IKKβ	4KIK	Kinase domain (homodimeric structure)	x=47.179, y=30.100, z=−61.887	40×40×40	0.375
NLRP3	7PZC	NACHT domain	x=192.793, y=205.417, z=119.222	40×40×40	0.375
RELA (NF-κB p65)	1LE9	DNA-binding interface of RELA subunit (p50/p65 heterodimer structure)	x=117.674, y=18.111, z=19.444	40×40×40	0.375

For ADAM17, IKKβ, and NLRP3, docking grids were defined around the co-crystallized ligand/inhibitor binding sites on the relevant chain after ligand removal. β-Tubulin docking with its co-crystallized colchicine ligand served as an internal positive control.

5. Pose Selection and Interaction Analysis

For each receptor–ligand pair, at least 50 docking runs were performed using the Lamarckian Genetic Algorithm implemented in AutoDock. The resulting conformations were clustered based on RMSD criteria, and the top-ranked poses within the largest clusters were selected for further structural inspection. Docked conformations were ranked according to predicted binding free energy (ΔGb, kcal/mol). Conformations showing the lowest binding energy and clustering RMSD ≤ 2 Å were considered stable docking solutions. RMSD values refer to clustering RMSD obtained from the AutoDock docking output. Hydrogen bond interactions and residue contacts were evaluated using AutoDockTools and visualized using Python Molecular Viewer (PMV 1.5.6).

6. Reproducibility Statement

- All docking parameters were kept constant across targets to minimize methodological variability.
- The use of experimentally ligand-bound structures for β-tubulin served as an internal validation confirming accurate recovery of known binding orientations. Redocking of the co-crystallized colchicine ligand in the β-tubulin structure (PDB ID: 4O2B) reproduced the experimental binding pose with an RMSD below 2 Å, supporting the reliability of the docking protocol.
- Only reproducible interactions consistent across independent docking runs were included.
- No post-hoc score modifications or selective exclusions were applied.

7. Computational Validation Strengthening

- Convergence-based assessment strategies were used, including pose clustering consistency, interaction reproducibility across independent docking runs, and structural plausibility within experimentally characterized binding pockets.
- Predicted residues were compared with literature-reported functional regions to prevent overinterpretation.
- Docking outcomes were interpreted as hypothesis-generating mechanistic evidence complementary to experimental histomorphometric and histopathological findings.

8. Pharmacological Interpretation Framework

- Binding affinity scores were not considered standalone indicators of biological activity.
- Ligand orientation consistency and alignment with known functional regions informed interpretation.
- Hydrogen bonds, hydrophobic stabilization, and electrostatic complementarity were prioritized for biological plausibility.
- Only docking poses consistent with structural and functional constraints were incorporated, reducing false-positive inference.
- Computational results support mechanistic hypotheses but do not imply direct causal confirmation.

9. Reporting Consistency

This supplementary workflow corresponds directly to the following sections in the main manuscript:

- Section 2.5 (Molecular Docking and GO Enrichment Analysis)
- Section 3.3 (Docking Analysis Results)

All reported parameters, docking criteria, and analytical decisions are fully aligned with the methods described in the primary article to ensure transparency and reproducibility.

10. Reproducibility Statement (Data and Parameter Transparency)

All computational procedures were conducted using explicitly defined parameters and standardized workflows. Protein structures were obtained from publicly accessible structural databases. Ligand preparation followed uniform preprocessing steps including geometry optimization, hydrogen addition, and charge assignment using the same software environment.

Docking grid dimensions, search parameters, scoring functions, and pose selection criteria were applied consistently across all ligand–target pairs without manual optimization. Default algorithmic settings were preserved unless explicitly justified. All analyses were performed under identical computational conditions to maintain comparability.

Output evaluation criteria, including pose ranking, RMSD thresholds, and interaction assessment procedures, were predefined prior to interpretation. No selective exclusion or post-hoc adjustment of docking results was performed.

Supplementary Results and Additional Data

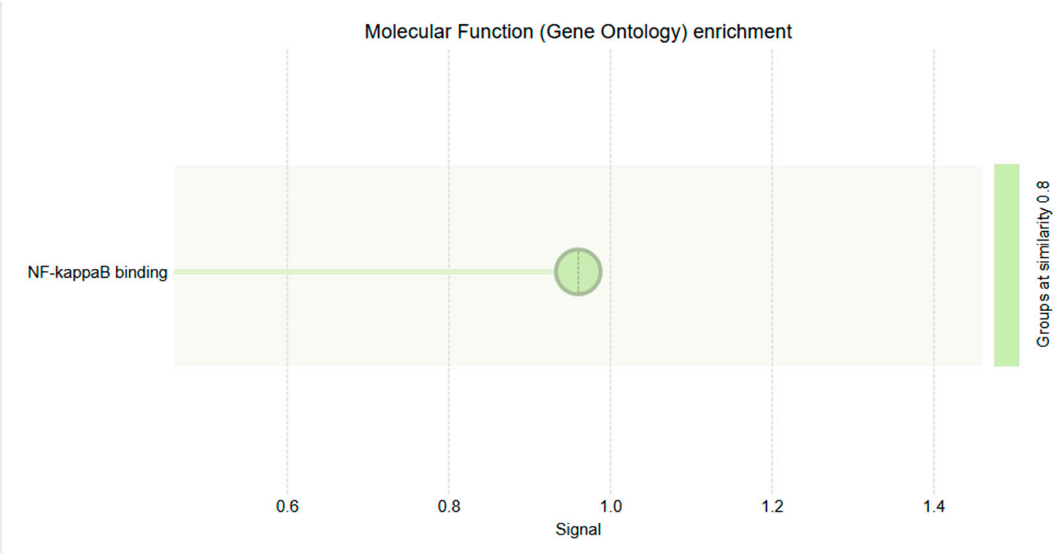

Figure S1. Molecular Function (MF) Gene Ontology enrichment analysis of the selected target genes. Terms are ranked based on adjusted p-values, and the network visualization reflects functional similarity clustering.

Table S2. Longitudinal Peripheral Blood Parameters in Control and Colchicine-Treated Groups

Day	Group	TNF-α (pg/mL)	IL-1β (pg/mL)	CRP (mg/L)	WBC (10^3/μL)	MDA (nmol/mL)	SOD (U/mL)
7	Control	42.3 ± 8.1	38.7 ± 7.4	12.4 ± 2.8	9.8 ± 1.4	4.21 ± 0.58	70.8 ± 9.6
	Colchicine	40.1 ± 7.9	36.9 ± 7.1	11.8 ± 2.6	9.4 ± 1.3	4.14 ± 0.54	71.9 ± 9.2
14	Control	35.6 ± 7.3	32.4 ± 6.8	8.9 ± 2.1	8.2 ± 1.2	3.61 ± 0.48	83.4 ± 10.2
	Colchicine	34.2 ± 7.0	31.1 ± 6.5	8.4 ± 2.0	7.9 ± 1.1	3.52 ± 0.45	85.1 ± 10.6
28	Control	28.4 ± 6.2	25.8 ± 5.9	5.6 ± 1.6	7.1 ± 1.0	2.76 ± 0.42	100.7 ± 11.1
	Colchicine	27.1 ± 5.9	24.6 ± 5.6	5.2 ± 1.5	6.8 ± 0.9	2.71 ± 0.40	101.8 ± 11.5
p (time effect)		0.63	0.71	0.14	0.09	<0.01*	<0.01*

Values are expressed as mean ± SD (n = 10 per group per time point). No statistically significant between-group differences were detected for any parameter at any time point ($p > 0.05$, Mann–Whitney U test).

A significant time-dependent effect was observed for MDA and SOD (* $p < 0.01$, linear mixed-effects model); no significant group-by-time interaction was found. WBC: white blood cell count; CRP: C-reactive protein; MDA: malondialdehyde; SOD: superoxide dismutase; TNF-α: tumor necrosis factor-alpha; IL-1β: interleukin-1 beta.
